# Interpretable Artificial Intelligence Analysis of Functional Magnetic Resonance Imaging for Migraine Classification: Quantitative Study

**DOI:** 10.2196/72155

**Published:** 2025-09-03

**Authors:** Guohao Li, Hao Yang, Li He, Guojun Zeng

**Affiliations:** 1 West China Hospital, Sichuan University Chengdu China; 2 Division of Vascular Surgery Department of General Surgery West China Hospital, Sichuan University Chengdu China

**Keywords:** migraine, personalized diagnosis, convolutional neural networks, explainable artificial intelligence, XAI, gradient-weighted class activation mapping

## Abstract

**Background:**

Deep learning has demonstrated significant potential in advancing computer-aided diagnosis for neuropsychiatric disorders, such as migraine, enabling patient-specific diagnosis at an individual level. However, despite the superior accuracy of deep learning models, the interpretability of image classification models remains limited. Their *black-box* nature continues to pose a major obstacle in clinical applications, hindering biomarker discovery and personalized treatment.

**Objective:**

This study aims to investigate explainable artificial intelligence (XAI) techniques combined with multiple functional magnetic resonance imaging (fMRI) indicators to (1) compare their efficacy in migraine classification, (2) identify optimal model-indicator pairings, and (3) evaluate XAI’s potential in clinical diagnostics by localizing discriminative brain regions.

**Methods:**

We analyzed resting-state fMRI data from 64 participants, including 21 (33%) patients with migraine without aura, 15 (23%) patients with migraine with aura, and 28 (44%) healthy controls. Three fMRI metrics—amplitude of low-frequency fluctuation, regional homogeneity, and regional functional connectivity strength (RFCS)—were extracted and classified using GoogleNet, ResNet18, and Vision Transformer. For comprehensive model comparison, conventional machine learning methods, including support vector machine and random forest, were also used as benchmarks. Model performance was evaluated through accuracy and area under the curve metrics, while activation heat maps were generated via gradient-weighted class activation mapping for convolutional neural networks and self-attention mechanisms for Vision Transformer.

**Results:**

The GoogleNet model combined with RFCS indicators achieved the best classification performance, with an accuracy of >98.44% and an area under the receiver operating characteristic curve of 0.99 for the test set. In addition, among the 3 indicators, the RFCS indicator improved accuracy by approximately 8% compared with the amplitude of low-frequency fluctuation. Brain activation heat maps generated by XAI technology revealed that the precuneus and cuneus were the most discriminative brain regions, with slight activation also observed in the frontal gyrus.

**Conclusions:**

The use of XAI technology combined with brain region features provides visual explanations for the progression of migraine in patients. Understanding the decision-making process of the network has significant potential for clinical diagnosis of migraines, offering promising applications in enhancing diagnostic accuracy and aiding in the development of new diagnostic techniques.

## Introduction

### Background

Migraines are a common, incapacitating neurovascular disorder characterized by attacks of severe headaches and autonomic nervous system dysfunction [1,2]. Diagnosing migraines, in general, is a complex task due to the subjective nature of the condition [3]. The symptoms of patients vary greatly, often overlapping with those of other neurological or medical diseases, and the lack of distinct biomarkers or image characteristics also increases diagnostic challenges. Furthermore, migraine with aura (MWA) and migraine without aura (MWoA) are the 2 primary types of migraines [4]. The main challenge in distinguishing between these categories lies in the fact that the warning signs of MWA do not always manifest and vary considerably in duration and severity from person to person [5,6].

In recent years, the study of magnetic resonance imaging (MRI) has greatly advanced our understanding of the neural mechanisms underlying migraines [7], using blood oxygenation level–dependent (BOLD) signals to measure neural activity [8]. Most related findings were obtained by applying mass-univariate analysis techniques to detect group differences and probe the pathogenesis of migraines [9]. Three commonly used indicators—amplitude of low-frequency fluctuation (ALFF), regional homogeneity (ReHo), and regional functional connectivity strength (RFCS)—can effectively help us analyze functional magnetic resonance imaging (fMRI) data because they reflect brain activity characteristics from different perspectives. As a result, these metrics have been widely applied in neuropsychiatric research and have proven valuable in uncovering the neural mechanisms of diseases [10-12]. For example, compared with healthy controls (HCs), patients with migraines have been shown to exhibit significant ALFF variations in the bilateral middle occipital cortex or cuneus and ReHo changes in the prefrontal cortex, orbitofrontal cortex [13], insula [14], and cuneus [15]. Altered functional connectivity (FC) has also been identified between the dorsolateral prefrontal cortex and the dorsal anterior cingulate cortex [16]. However, this analysis lacks a personalized diagnosis for patients in a clinic setting.

Recent studies have highlighted the potential of machine learning in migraine classification; however, challenges such as inconsistent study design and lack of methodological transparency underscore the need for robust frameworks such as deep learning to improve generalization and clinical applicability [17]. Deep learning has been applied to medical data across a variety of fields, allowing for inferences at the level of individual participants and thus solving this problem. Furthermore, it is sensitive to subtle and spatially distributed differences in the brain; excels in automatically extracting intermediate and high-level feature representations from raw data; and identifies the crucial features necessary for accurate classification, which might be undetectable in group comparisons. Ashina et al [18] further emphasized that even though deep learning models demonstrate superior accuracy, their “black box” nature remains a critical barrier to achieving biomarker discovery and personalized treatment. Therefore, developing deep learning frameworks that integrate both high accuracy and interpretability is essential for deciphering the heterogeneous mechanisms of migraine and advancing precision medicine [18].

### Objectives

We hypothesize that deep learning models can classify patients with migraine versus HCs using resting-state fMRI (rs-fMRI) data with higher accuracy than traditional methods, while explainable techniques will reveal distinct neurofunctional patterns that align with known migraine-related pathophysiology, thereby bridging the “black box” gap. In this research, we used an array of deep learning architectures, including GoogleNet [19] and ResNet [20], and integrated the self-attention mechanism of the Vision Transformer (ViT) [21] model, alongside various modalities of MRI data for classification purposes. These deep learning architectures each have their own advantages and can extract features from rs-fMRI data from different perspectives. Moreover, these models have been widely applied in medical image analysis and proven effective [22]. We implemented the gradient-weighted class activation mapping (Grad-CAM) [23] technique for convolutional neural networks (CNNs). This method significantly enhances the interpretability of the classification results, thereby increasing the model’s credibility in the context of migraine diagnosis. For the ViT model, we leveraged the attention mechanism to improve interpretability by visualizing attention weights. This allows us to visually identify the regions of the image that the model focuses on when making classification decisions. By using these explainable artificial intelligence (XAI) [24] techniques and visualizing attention maps, we can delineate the approximate locations of the regions of interest via a heat map, thus facilitating the interpretation of the classification results generated by the deep learning models.

## Methods

### Participants

This study enrolled 64 individuals, categorized into 3 groups: 21 (33%) patients with MWoA, 15 (23%) patients with MWA, and 28 (44%) HC. The patients were recruited from the internal medicine–neurology department of West China Hospital, Sichuan University, and diagnoses were confirmed by neurologists specializing in headache disorders based on the International Classification of Headache Disorders criteria.

Participants were right-handed adults aged 18 to 50 years. They discontinued analgesic medications for ≥2 weeks and other medications for ≥1 month before the study, with no ongoing prophylactic treatment. All patients remained migraine free for at least 72 hours before the brain scan and throughout the 48-hour follow-up period after scanning. Exclusion criteria included for the HCs, and inclusion criteria required no personal or family history of migraine or other headache disorders and no history of neuropsychiatric disorders or neurological impairments. Controls were age matched (±7 y) and sex matched to patients. Exclusion criteria for all groups included MRI contraindications, substance abuse history, and neurodevelopmental disorders. HCs underwent additional screening via structured clinical interviews to exclude headache disorders.

It is important to note that while the presence of a clinical diagnosis of neuropsychiatric disorders was an exclusion criterion, subclinical levels of depression and anxiety were not. The inclusion of participants with varying degrees of depression and anxiety allowed us to investigate the natural variability of these conditions within the migraine population and HCs. Exclusion criteria for both patient and control groups included the presence of chronic migraines, concurrent pain conditions, history of neuropsychiatric disorders, or any other neurological impairments that could affect the imaging results.

Demographic data (age, sex, and education) and psychometric scores (Hamilton Depression Scale and Hamilton Anxiety Scale) were collected during initial screening using standardized report forms. Psychometric evaluations revealed varied levels of depression and anxiety among the groups, quantified by the Hamilton Depression Scale and Hamilton Anxiety Scale.

### Ethical Considerations

The research was approved by the institutional review board of West China Hospital, Sichuan University (2020-666; Figure S1 in Multimedia Appendix 1). All participants provided written informed consent, including explicit permission for secondary data analysis. Participants were informed about the study’s purpose, data use, and privacy protections. The consent forms allowed future reuse of anonymized data. All data were deidentified before analysis, with Digital Imaging and Communications in Medicine header information removed using specialized tools. No identifiable facial features or personal metadata were present in any of the presented images.

### Statistical Analysis

This study used the Kruskal-Wallis *H* test from the *scipy.stats* package of Python (Python Software Foundation) to analyze the experimental data. This is a nonparametric test used to determine whether there are statistically significant differences in medians among ≥3 independent samples [25].

The specific method involves combining all data from the 3 sample types used in this study and ranking them by value, assigning each data point a corresponding rank. If values are tied, the average rank is calculated. Next, the rank sums (R1, R2, and R3) for each sample group are computed separately. The *H* statistic is then calculated using the Kruskal-Wallis *H* test formula:




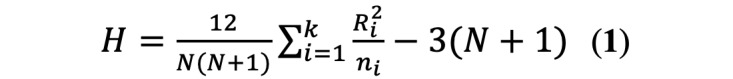




where *N* represents the total sample size across all groups, *k* represents the number of groups (in this case, *k*=3), *n*_i_ represents the sample size of the *i*-th group, and *R*_i_ represents the rank sum of the *i*-th group.

Finally, the calculated *H* value is compared with the chi-square distribution with 2 *df*sto obtain the corresponding *P* value. If *P*≤.05, it indicates that at least 1 of the 3 sample groups has a median significantly different from the others. If *P*>.05, there is insufficient evidence to conclude a significant difference in medians among the 3 sample groups.

### Neuroimaging Data Acquisition and Preprocessing

Data acquisition was performed using a 3.0 Tesla MRI system (Trio Tim, Siemens). Participants were instructed to rest with their eyes closed, remain awake, and avoid active thinking. Structural imaging was conducted using a transverse echoplanar imaging sequence with the following parameters: the ratio of repetition time to echo time (TR/TE)=2000/30 ms, flip angle=90°, slice thickness/gap=5/0 mm, field of view =240×240 mm², matrix=64×64, and voxel size=3.75×3.75×5 mm³. The scanning duration for all participants was 6 minutes (360 s), corresponding to 180 time points.

We preprocessed the data using the Graph Theoretical Network Analysis toolbox and Statistical Parametric Mapping. The preprocessing steps included the following:

- The first 10 time points were removed from each functional dataset to account for initial signal instability and participant adaptation, leaving 170 time points (340 s) for subsequent processing
- Slice timing correction and realignment were performed to minimize motion artifacts. Participants with head motion exceeding 2-mm displacement or 2° rotation were excluded. All participants in this study met these motion criteria.
- Spatial normalization was conducted to standard Montreal Neurological Institute space with 3-mm isotropic voxels
- Bandpass filtering (0.01-0.08 Hz) was applied to each voxel’s time series to reduce low-frequency drift and high-frequency physiological noise [26]
- Spatial smoothing was conducted using a 4-mm full width at half maximum Gaussian kernel.
- Regression of nuisance covariates was performed to mitigate the influence of nonneuronal signals.

Finally, the ALFF, ReHo, and RFCS were calculated. ALFF serves as a reliable metric of regional intrinsic neuronal activity [27]. Time series from each voxel were converted to the frequency domain via fast Fourier transform to derive the power spectrum. The square root of the power spectrum was then averaged over a predefined frequency range. Within the 0.01 to 0.08 Hz frequency band, ALFF was calculated per voxel using the rs-fMRI Data Analysis Toolkit (REST, Provided by the REST team led by Professor ZANG Yu-Feng, ORCID: 0000-0003-1833-8010) software. To control for interparticipant variability, the ALFF values of individual voxels were normalized by dividing them by the global mean ALFF. ReHo, quantified using the Kendall coefficient of concordance, measures the temporal similarity between a voxel and its immediate neighbors, yielding consistent outcomes in rs-fMRI analyses. In our study, a cubic cluster of 27 voxels was defined for each normalized and resliced image, with the ReHo value of each cluster attributed to the central voxel. Higher ReHo values indicate greater local synchronization of rs-fMRI signals among adjacent voxels. Similar to ALFF normalization, each voxel’s ReHo value was divided by the global mean ReHo value per participant. These procedures were carried out using REST software.

The rs-fMRI enables the evaluation of brain function through the measurement of FC among distinct brain regions. To mitigate the impact of regions of interest selection on FC outcomes, we adopted the RFCS approach, which evaluates the average correlation between a given brain region and all others [28]. To calculate resting-state FC, we controlled for the spurious effects of nuisance covariates [29]. The rs-fMRI data were parcellated into 116 regions of interest based on the automated anatomical labeling (AAL) template, yielding 116 FC results for each participant. The RFCS values were subsequently determined using a methodology detailed in a previous study by Jiang et al [30]. The RFCS was defined as follows:




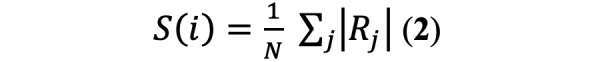




The preprocessed neuroimaging data were managed using the NiBabel library, converting 4D data into 2D matrices suitable for CNNs. Throughout the data conversion process, MRI scans from all 64 participants were processed. Each scan initially produced 33 slices; the first 3 slices were discarded due to potential quality issues, leaving 30 valid slices per participant. Each slice then underwent 3 feature mappings, resulting in a total of 5760 images: 1890 from patients with MWoA, 1350 from patients with MWA, and 2520 from HCs. We ensured that no slices from the same patient appeared in both the training and testing sets to avoid data leakage. To mitigate overfitting, data augmentation techniques, such as cropping, rotating, and flipping of input images, were implemented [31,32]. Further data augmentation, involving random shuffling and resizing to 224×224 pixels, increased the diversity of the dataset. The entire training phase was conducted on a high-performance GPU (Nvidia 4070 Ti with CUDA support), using the PyTorch framework for the implementation of the deep learning model.

### Model Validation

For the machine learning applications, we first performed a 5-fold cross-validation to split the participants’ data into training and testing sets. Specifically, the entire dataset is first randomly divided into 5 roughly equal parts, a step commonly referred to as folding. Next, 5 iterations are performed. In each iteration, 1 fold is selected as the test set, while the remaining 4 folds are combined to form the training set. The model is trained on the training set and evaluated on the test set, with the evaluation results recorded. After all 5 iterations are completed, the results of the 5 evaluations are averaged to obtain the final model performance assessment. This averaged value provides a more stable and reliable reflection of the model’s performance on the data.

In addition, we also used leave-one-out cross-validation (LOOCV) methodology for comparative validation purposes.

### Deep Learning Architecture

CNN architectures include several CNN layers that transform an input image step by step, ultimately yielding a class prediction. This study examines some of the most popular CNN architectures used for image recognition tasks, including GoogleNet and ResNet18. In contrast, the ViT is a transformer-based architecture that divides an image into multiple small patches, treating these patches as elements in a sequence, and processes them through the Transformer model. This approach leverages self-attention mechanisms to capture global contextual information within the image. Compared with traditional CNNs, ViT offers greater flexibility and scalability in image processing. The introduction of ViT has brought a new perspective to the field of image recognition, achieving remarkable performance on various benchmark tests. These different models were included to test the impact of network architecture on visualization performance. Results derived from each model were compared, and the best-performing algorithm was used as the basis for visualization.

In addition, to provide a more comprehensive performance comparison, we included 2 widely used traditional machine learning methods—support vector machine and random forest—as baseline models, performing 3-class classification directly using features extracted from RFCS.

### Transfer Learning

Currently, optimization algorithms such as Stochastic Gradient Descent [33] have significantly improved our training efficiency. To further enhance model performance and reduce training time, we also used transfer learning. Transfer learning increases a model’s robustness and accuracy when performing new classification tasks by leveraging knowledge acquired from related tasks, which is particularly beneficial for small training datasets [34]. Initialization weights that have been trained to recognize various items in different images are easier to train than initial random weights, as image recognition primarily involves detecting combinations of edges. The initial weights used in our model included transfer learning weights from the ImageNet competition, derived from images with similar features. In addition, all weights throughout the network were frozen and did not participate in backpropagation, except for those in the last convolutional layer and the fully connected layer. These layers were replaced with new layers exhibiting random weights. By training only the updated layers, we reduced runtime by limiting the number of calculations required in each forward pass iteration. This approach also improved classification performance by simplifying the model and reducing the number of parameters.

### XAI Methods

XAI methods are designed to enhance the transparency of machine learning models, enabling a clear understanding of the models’ decision-making processes. XAI is particularly well suited for applications that demand a high level of trust and safety. Among the commonly used interpretability techniques are Grad-CAM and the visualization of self-attention mechanisms.

Grad-CAM merges the gradients of target concepts with the final convolutional layer to produce a coarse localization map, highlighting the important regions in an image for the purpose of predicting feature concepts. A previous study by Hao et al [35] has asserted that deeper representations of models allow for the capture of higher-level visual constructs. In addition, convolutional features naturally retain spatial information, which is lost in fully connected layers. As such, we can expect the last convolutional layer to exhibit the best compromise between high-level semantics and detailed spatial information. Grad-CAM can also be used to represent so-called counterfactual explanations, regions that (if removed) could change the classification results. Neurons in the latter layers of a CNN are mostly used to identify semantic class-specific information in an image (ie, specific structures). In contrast, Grad-CAM uses gradient information passed to the last convolutional layer of the CNN to understand the importance of each neuron for a decision of interest.

**Figure 1:**
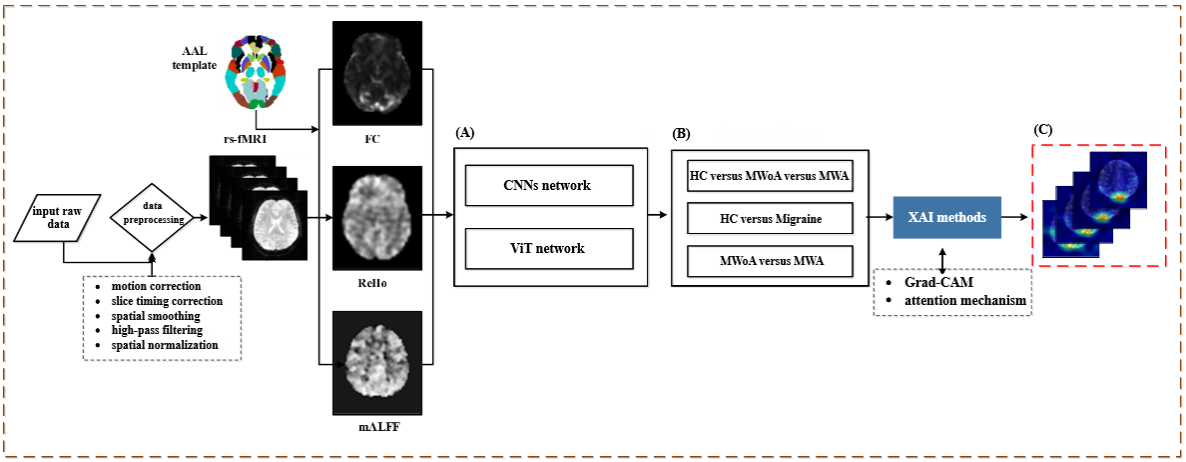
A schematic illustration of the proposed classification process. (A) Deep learning models; (B) Output layer; (C) Activation map. AAL: Automated Anatomical Labeling; CNN: convolutional neural network; FC: functional connectivity; Grad-CAM: gradient-weighted class activation mapping; HC: healthy control; mALFF: mean amplitude of low-frequency fluctuation; MWA: migraine with aura; MWoA: migraine without aura; ReHo: regional homogeneity; rs-fMRI: resting-state function magnetic resonance imaging; ViT: Vision Transformer; XAI: explainable artificial intelligence.

The ViT model enhances its ability to capture image features by dividing the image into multiple patches and using the self-attention mechanism [36] in the Transformer architecture. This approach captures the relationships and global contextual information between different regions of the image. By visualizing the attention weights, we can intuitively see which areas of the image the model focuses on when making classification decisions. This visualization of attention maps provides a clear method for understanding the model’s decision-making process, thereby improving its interpretability. This enhanced understanding of how the model identifies and distinguishes different categories of images is crucial for increasing the model’s transparency and credibility. A flowchart for this process is shown in Figure 1.

### Evaluation Criteria

#### Receiver Operating Characteristic Curve

Prediction model performance was evaluated for the test sets using a receiver operating characteristic (ROC) curve [37]. The area under the curve (AUC) is a quantitative metric assessing the overall performance of a binary classifier without setting a specific threshold. AUC ranges from 0 to 1, where an AUC of 1 indicates perfect classification, and an AUC of 0.5 suggests the model performs no better than random guessing. Values <0.5 are uncommon in practice, as reversing the classification would improve results. Higher AUC values (>0.5) indicate better class separation.

To comprehensively evaluate the classification performance, both AUC and the *F*_1_-score were used as key performance indicators. AUC measures the model’s ability to distinguish between classes across all thresholds, making it robust to class imbalance and useful when misclassification costs vary between classes. The *F*_1_-score, defined as the harmonic mean of precision and recall, provides a balanced measure especially suitable for imbalanced datasets where both false positives and false negatives are of concern [38]. The relevant metrics are defined as follows:




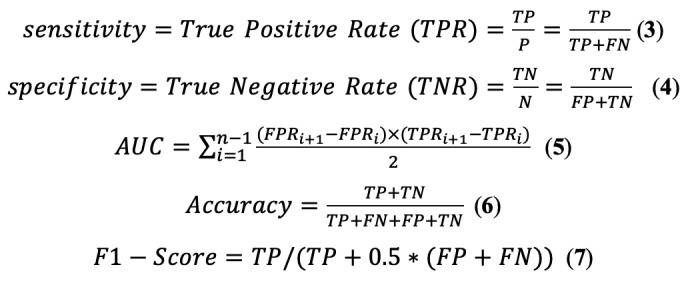




where *TP*, *FP*, *TN*, and *FN* represent true positive, false positive, true negative, and false negative, respectively.

#### Confusion Matrix

An analysis table can be used to summarize prediction results for a classification model and is particularly beneficial for multiclass objects (ie, distinguishing MWoA, MWA, and HC). In this process, decisions for specific data are summarized in a matrix form, using the real category and classification judgments made by the model. A confusion matrix was applied in this study to quantify the consistency between predicted and actual results.

## Results

### Classification Performance

Table 1 presents the research data, demonstrating that the observed differences in psychometric assessments were not confounded by demographic variables, such as sex, age, or education level.

**Table 1:** Baseline characteristics of patients with migraine^a^.

Variables		MWoA^b^	MWA^c^	HC^d^	*P* value	
**Sex, n (%)**						.40
	Male	7 (11%)	4 (6%)	13 (20%)		
	Female	14 (22%)	11 (17%)	15 (24%)		
Age (y), median (IQR)		29.00 (26.00-31.00)	29.00 (27.00-35.00)	29.00 (27.00-34.25)	.47	
Education (y), median (IQR)		19.00 (16.00-19.00)	16.00 (15.00-16.00)	16.00 (16.00-19.00)	.002	
24-HAMD^e^, median (IQR)		4.00 (1.00-9.00)	10.00 (6.00-15.00)	1.00 (0.00-3.00)	<.001	
14-HAMA^f^, median (IQR)		3.00 (1.00-6.00)	6.00 (5.00-9.50)	0.00 (0.00-3.00)	<.001	

^a^Statistical differences between groups were calculated using the Kruskal-Wallis test.

^b^MWoA: migraine without aura.

^c^MWA: migraine with aura.

^d^HC: healthy control.

^e^HAMD: Hamilton Depression Scale.

^f^HAMA: Hamilton Anxiety Scale.

Classification performance was tested using deep learning models with different rs-fMRI indicators (ALFF, ReHo, and RFCS). The data were divided into 2 groups before being input to the model: migraine versus HC and MWA versus MWoA. The loss of training and testing data were measured during the training phase. All models exhibited an improved classification accuracy (>84%) when compared with conventional machine learning methods applied to the same data. Our group previously used the LOOCV technique to obtain higher ranking features from the square of a weight vector coefficient, used as a ranking criterion to determine features for training a multicore support vector machine classifier [39].

**Figure 2:**
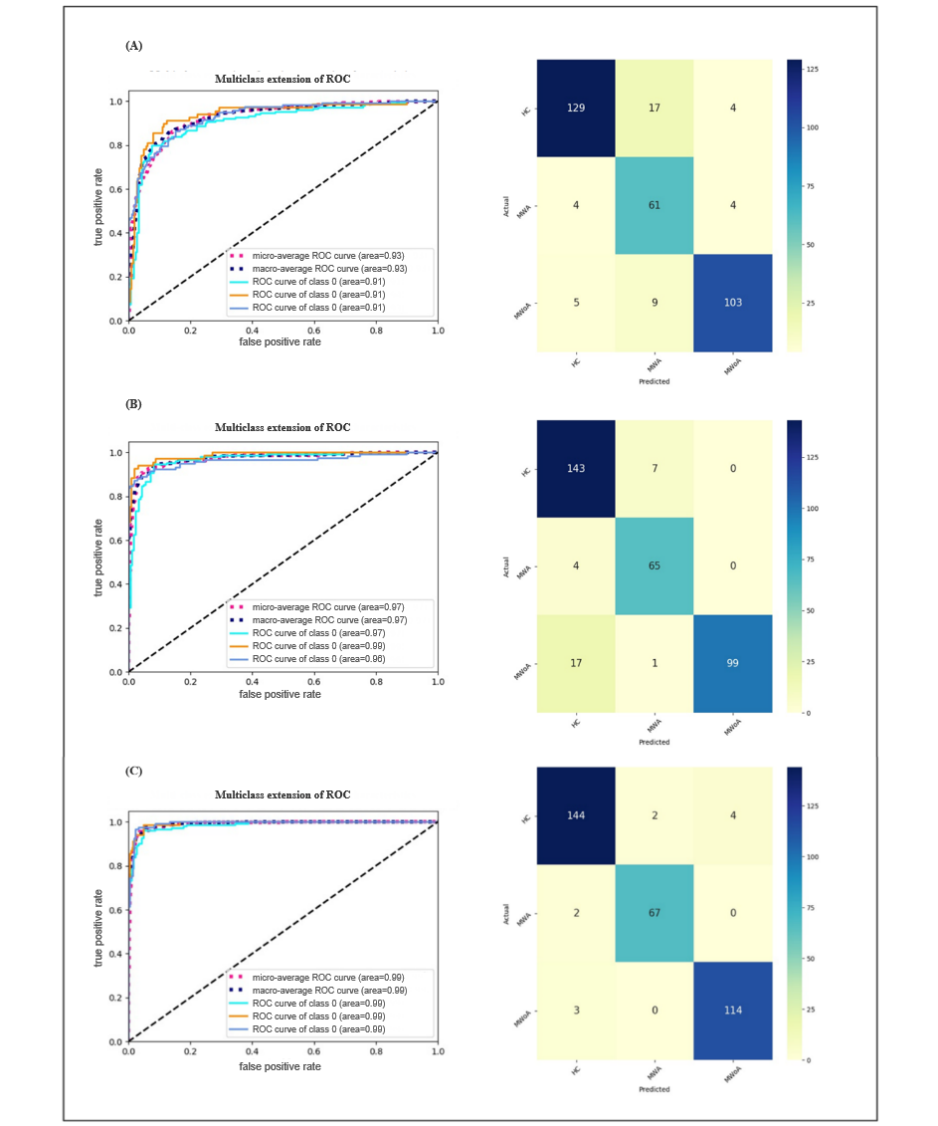
Performance classification as determined by various indicators. (A) The ALFF indicator as input to GoogleNet; (B) The ReHp indicator as input to GoogleNet; (C) The RFCS indicator as input to GoogleNet. ALFF: amplitude of low-frequency fluctuation; HC: healthy control; MWA: migraine with aura; MWoA: migraine without aura; ReHo: regional homogeneity; RFCS: regional functional connectivity strength; ROC: receiver operating characteristic curve.

Confusion matrices and ROC curves were also calculated for each model and used to display the classification results. We found that GoogleNet produced the highest classification accuracy for a given set of indicators, as shown in Figure 2.

In addition, RFCS achieved the best performance for each classifier among the tested indicators. Therefore, GoogleNet combined with RFCS provided the highest average precision for identifying patients with migraine. In the results, we observed that the use of the ViT-B/16 model did not yield an improvement in accuracy. In fact, compared with the straightforward CNN architecture, the classification outcomes of the ViT model showed a decrease in performance across various MRI modalities. The ultimate accuracy of the models was then determined by averaging the accuracies from 4 distinct trials, as illustrated in Table 2. In addition, we performed LOOCV using the GoogleNet model that achieved the best classification performance, with results available in Table S1 in Multimedia Appendix 1.

**Table 2:** Results of 5-fold cross-validation based on 3 metrics across different deep learning models.

Model, indicator, and data group				Accuracy (%), mean (SD)		*F*_1_-score (%), mean (SD)
**ResNet18**						
	**ALFF^a^**					
		HC^b^ versus migraine	87.20 (0.64)		87.13 (0.62)	
		HC versus MWoA^c^ versus MWA^d^	86.83 (1.84)		86.70 (1.85)	
	**ReHo^e^**					
		HC versus migraine	92.56 (0.94)		92.50 (0.90)	
		HC versus MWoA versus MWA	91.07 (0.37)		90.95 (0.35)	
	**RFCS^f^**					
		HC versus migraine	97.81 (0.39)		97.7 (0.40)	
		HC versus MWoA versus MWA	96.75 (1.93)		96.55 (1.95)	
**GoogleNet**						
	**ALFF**					
		HC versus migraine	90.69 (1.12)		90.50 (1.10)	
		HC versus MWoA versus MWA	89.95 (0.65)		89.8 (0.65)	
	**ReHo**					
		HC versus migraine	94.79 (1.29)		94.6 (1.30)	
		HC versus MWoA versus MWA	93.52 (0.51)		93.4 (0.50)	
	**RFCS**					
		HC versus migraine	98.71 (0.14)		98.65 (0.15)	
		HC versus MWoA versus MWA	98.44 (0.29)		98.3 (0.30)	
**ViT-B/16^g^**						
	**ALFF**					
		HC versus migraine	84.58 (1.83)		84.03 (0.67)	
		HC versus MWoA versus MWA	84.51 (0.56)		83.95 (0.60)	
	**ReHo**					
		HC versus migraine	87.78 (0.61)		87.3 (0.65)	
		HC versus MWoA versus MWA	86.7 (1.27)		86.1 (1.30)	
	**RFCS**					
		HC versus migraine	93.04 (0.50)		92.8 (0.50)	
		HC versus MWoA versus MWA	92.93 (1.25)		92.6 (1.30)	

^a^ALFF: amplitude of low-frequency fluctuation.

^b^HC: healthy control.

^c^MWoA: migraine without aura.

^d^MWA: migraine with aura.

^e^ReHo: regional homogeneity.

^f^RFCS: regional functional connectivity strength.

^g^ViT: Vision Transformer.

**Figure 3:**
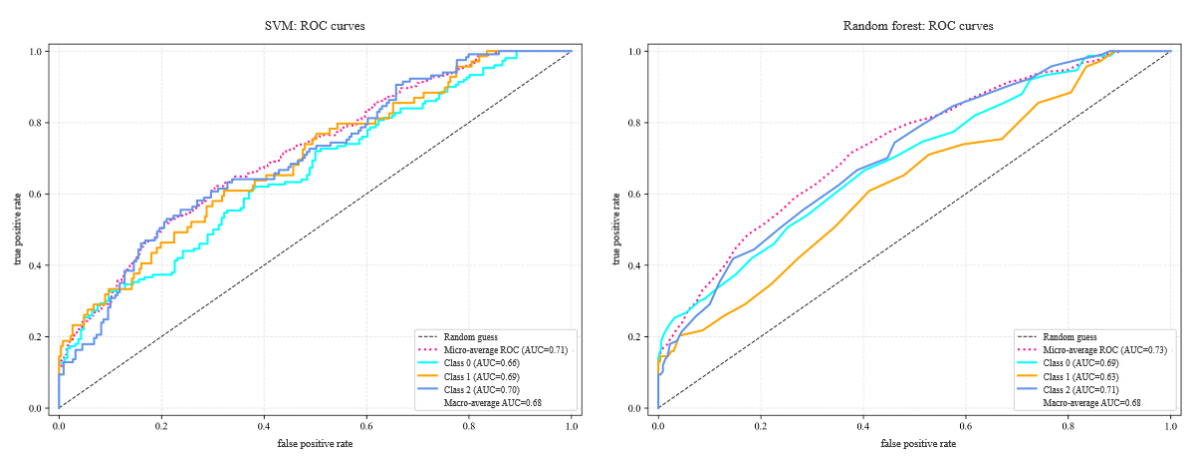
The regional functional connectivity strength (RFCS) indicator as input to support vector machine (SVM) and random forest. AUC: area under the curve; ROC: receiver operating characteristic curve.

The ROC curves are shown in Figure 3, and the classification results are presented in Table 3.

**Table 3:** Three-class classification performance of support vector machine (SVM), random forest (RF), and deep learning models using regional functional connectivity strength (RFCS) features.

Metric: HC^a^ versus MWoA^b^ versus MWA^c^	SVM: accuracy (%), mean (SD)	RF: accuracy (%), mean (SD)	GoogleNet: accuracy (%), mean (SD)
Accuracy	50.71 (1.65)	53.45 (1.12)	98.44 (0.29)
Precision	47.7 (2.08)	60.48 (2.96)	86.44 (0.26)
Recall	46.74 (1.89)	45.27 (1.19)	84.1 (0.32)
*F*_1_-score	46.91 (2.00)	44.08 (1.50)	98.3 (0.30)
AUC^d^	66.29 (1.47)	66.9 (0.57)	98.63 (0.19)

^a^HC: healthy control.

^b^MWoA: migraine without aura.

^c^MWA: migraine with aura.

^d^AUC: area under the curve.

### Model Visualization

Grad-CAM was used to visualize the GoogleNet model that achieved the best classification performance across various tasks. This process, representing the first time that CNN visualization of migraine classification results has been conducted using different MRI indicators, can be described in several steps. First, backpropagation was performed from the correctly classified images of patients with migraine. The mean gradient in each channel was then determined and multiplied by postactivated values in the feature map. Second, the absolute values of the gradient-weighted feature maps were summed to produce a coarse localization map, which was then thresholded and resized to the original image resolution. This localization map was converted into a heat map by applying a color scale to identify specific brain regions involved in classification tasks.

**Figure 4:**
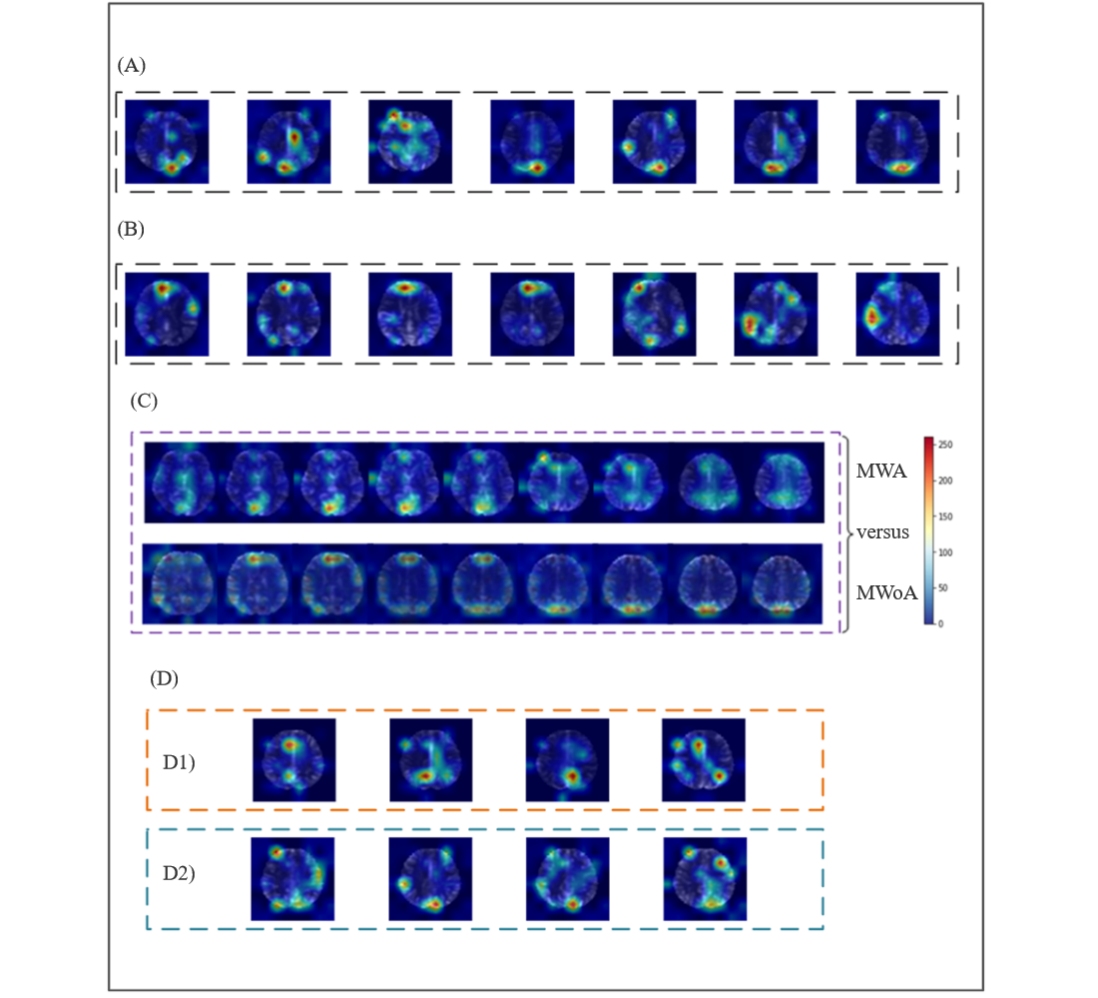
Activation heat maps derived from amplitude of low-frequency fluctuation (ALFF) features. (A) Activation heatmaps for multiple slices from a patient with MWA. (B) Activation heatmaps for multiple slices from a patient with MWoA. (C) Mean activation heatmaps for MWA versus MWoA. (D) Activation heatmaps for incorrectly classified images; (D1) HC samples incorrectly classified as patient with migraine; (D2) MWA samples incorrectly classified as MWoA. HC: healthy control; MWA: migraine with aura; MWoA: migraine without aura.

**Figure 5:**
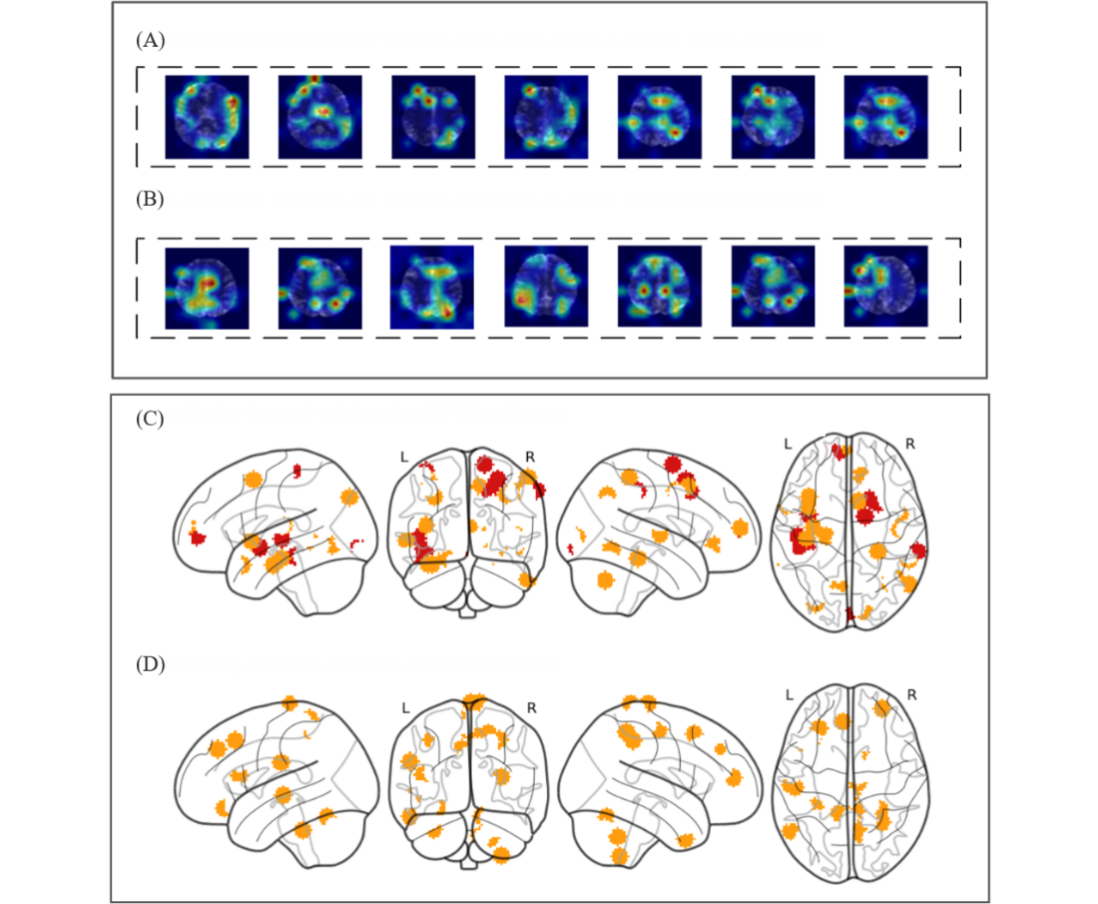
Activation heat maps derived from regional homogeneity (ReHo) and regional functional connectivity strength (RFCS) metrics, along with high-resolution activation heat maps based on RFCS, demonstrating the activation patterns across multiple brain regions. (A)Activation heatmaps for multiple slices from an MWA patient (ReHo indicator); (B) Activation heatmaps for multiple slices from an MWA patient (RFCS indicator). (C) Group 1: health control versus migraine (RFCS indicator); (D) Group 2: MWoA versus MWA (RFCS indicator). MWA: migraine with aura; MWoA: migraine without aura.

In this study, 3 MRI indicators—ALFF, ReHo, and RFCS—were registered and spatially mapped onto the AAL template to ensure accurate localization. Using these indicators, we generated heat maps to determine the areas of the brain that contributed most significantly to the classification performance. For example, the ALFFs that produced the most prominent heat maps are shown in Figure 4. Heat maps produced with ReHo and RFCS are shown in Figures 5A and 5B. In addition, we generated heat map results for the ViT model based on its self-attention mechanism. In the self-attention layers, the model calculates the relationship weights between each patch and all other patches in the sequence. By aggregating the attention weight matrices of each patch with others, we obtain the attention distribution of each patch within the image, thus creating the model’s heat map (Figure S2B in Multimedia Appendix 1). However, the heat map lacks distinct hot spots, which may be due to the similar attention weights across patches, leading to a uniform attention distribution. This uniformity in the heat map could also explain the reduced classification performance of the model.

In addition, to obtain clearer and more intuitive activation maps, we additionally visualized the RFCS indicator activation area on another enlarged and high-definition brain template to achieve more precise localization of brain activity, as shown in Figures 5C and 5D. In Figure 5, group 1 displays the most discriminative activations between HCs and patients with migraine, with red intensity indicating stronger activation, and group 2 highlights the most discriminative activation regions between MWA and MWoA.

## Discussion

### Principal Findings

This study used the XAI methods to interpret deep learning models’ decision-making processes and produce activation heat maps for class-discriminative regions in MRI slices. Various fMRI indicators were included as input data for the classification of migraines by a deep learning model. Results were compared for various MRI indicators (ALFF, ReHo, and RFCS) and different network architectures (GoogleNet, ResNet18, and ViT-B/16). The GoogleNet model combined with RFCS indicators produced the best classification performance (>98.44%). Heat maps confirmed Grad-CAM to be a promising visualization technique for the clinical diagnosis of migraines.

We compared 3 deep learning models based on different fMRI indicators. GoogleNet achieved the best results among the 3 models, producing a performance increase of 2% to 3% (AUC) for each indicator (Table 1). The inception module in GoogleNet could merge receptive fields of different sizes simultaneously with a concatenation filter, which improved classification performance [40]. It was evident in this study that the selection of indicators had a significant effect on classification results, with the RFCS indicator improving accuracy by approximately 8% compared with ALFF. RFCS measures the average correlation between a given brain region and other regions. This was often based on the AAL template-116 brain region [41], which contained important information about the whole brain to enhance accuracy rates. In contrast, ALFF was used to calculate the intensity of brain activity in a single voxel and included far fewer features than RFCS. This result suggested that the improvements in classification were partly the result of model selection and primarily a consequence of indicator preference. In reviewing the literature, we found that education level was not always directly associated with migraines [42]. Therefore, its ultimate impact on classification performance might be small.

### Comparison With Prior Work

A comparison of average localization maps with the AAL template suggested the most discriminative brain regions, for differentiating the migraine and HC groups, to be the cuneus and precuneus. There was also some slight activation present in the frontal gyrus. The most significant areas for distinguishing MWoA from MWA were the frontal gyrus and bilateral cuneus. These findings are in agreement with similar results from other studies. For example, Li et al [43] reported a significant decrease in ALFF in the bilateral middle occipital cortex and cuneus, when compared with HC. In addition, regions exhibiting decreased ALFF in patients with migraine included the bilateral cerebellum posterior lobe, left cerebellum anterior lobe, bilateral orbital cortex, middle frontal gyrus, bilateral occipital lobe, right fusiform gyrus, and bilateral postcentral gyrus [44]. There was no obvious pattern in the HC group, which suggests that pain is somewhat randomly distributed in normal cases.

Similarly, Farago et al [45] studied the low-frequency components of a BOLD signal (0.01-0.08 Hz) in MWoA and MWA groups [45]. Results showed the resting-state amplitude of BOLD fluctuations in the bilateral frontal regions to be higher in patients with MWA than in those with MWoA. This is in agreement with this study, as the cuneus is part of the visual cortex area of the brain [46].

Our study demonstrated the feasibility and clinical relevance of interpretable deep learning techniques in distinguishing between HCs and patients with migraine based on neuroimaging biomarkers. The identified patterns aligned with existing clinical knowledge: the cuneus, involved in visual processing, was associated with migraine as patients often exhibited light sensitivity and photophobia during attacks; the role of the precuneus in self-referential processing and pain modulation might explain the altered FC observed in patients with migraine; and dysregulation in the frontal cortex could be contributed to impaired pain inhibition and cognitive symptoms in chronic migraine [47].

Grad-CAM propagated gradients from the final convolutional layer to the input space, highlighting regions critical for category-specific predictions. The self-attention mechanism was particularly suitable for processing sequential data or data with complex dependencies. In migraine rs-fMRI data analysis, the self-attention mechanism helped the model identify key patterns of FC between different brain regions. In summary, our discovery of abnormal brain regions was supported by abundant literature. By leveraging interpretable models, we not only validated these biologically plausible findings but also provided clinicians with actionable insights. Our method might enhance the convenience of personalized diagnosis or targeted neural regulation therapy in certain scenarios. Therefore, in clinical translation, both methods alleviate the *black box* problem by providing visual explanations (heat maps), enabling clinicians to validate findings against established neurobiological knowledge, thereby increasing trust in model outputs for discovering potential biomarkers.

### Limitations

While deep learning–based diagnostic tools have advantages, incorrect decisions can adversely affect patients, leading to misdiagnoses or missed diagnoses. Inaccuracies in our model could result in patients with migraine not receiving timely and appropriate treatment or subject normal participants to unnecessary medical interventions. Therefore, we emphasize the importance of thorough validation before clinical application and suggest using this model as an adjunct rather than the sole diagnostic tool.

Certain limitations existed in this study that should be noted. First, the sample size was relatively small. During the training of the ViT model, overfitting occurred (Figure S2A in Multimedia Appendix 1). For some specific clinical scenarios, a more complex and deeper network does not necessarily yield the best results; instead, it is crucial to select an appropriate model based on the characteristics and scale of the dataset. This was addressed in the study by using data augmentation techniques (ie, rotation, reflection, and cropping in PyTorch) and adding a dropout layer before the fully connected layer to mitigate overfitting. Second, the heat maps exhibited relatively limited resolution, due to a size limit imposed by feature maps in the last convolutional layer. The architecture and class activation map algorithm could be optimized using higher-resolution medical images to produce larger-scale feature maps.

### Future Directions

Future research should aim to enhance the model’s resolution capabilities, consider other imaging modalities or biological markers, and explore the adversarial attack approach to enrich the data and improve the robustness of the results [48]. In addition, we are also seeking collaborations with similar studies to share datasets, though this process may take time due to the high costs of such data collection. Meanwhile, we are making efforts to acquire data from other hospitals, despite current challenges in obtaining external data. We plan to incorporate multicenter data to evaluate the model’s generalizability across different populations and MRI scanners. In addition, we will conduct more in-depth comparisons with neurologists’ diagnostic consistency to assess the model’s value in assisting clinical diagnosis. As for sex differences, our preliminary analysis did not reveal a significant impact on the model’s performance. Nonetheless, given the known variations in migraine prevalence and manifestation between sexes, further research is warranted to explore this aspect in greater detail.

### Conclusions

This study has demonstrated that XAI techniques, combined with fMRI-derived FC metrics, can achieve high classification accuracy in distinguishing patients with migraine from HCs while providing interpretable biomarkers. The GoogleNet model paired with RFCS emerged as the optimal framework, achieving exceptional performance (accuracy >98.44%; AUC=0.99) and outperforming conventional machine learning benchmarks. Generating heat maps by identifying the most discriminative brain regions also confirms that XAI using Grad-CAM is a promising visualization technique.

## Appendix

Multimedia Appendix 1 Ethical review supporting documentation and comparative experimental results.

## Abbreviations

AAL: automated anatomical labeling
ALFF: amplitude of low-frequency fluctuation
AUC: area under the curve
BOLD: blood oxygenation level–dependent
CNN: convolutional neural network
FC: functional connectivity
fMRI: functional magnetic resonance imaging
Grad-CAM: gradient-weighted class activation mapping
HC: healthy control
LOOCV: leave-one-out cross-validation
MRI: magnetic resonance imaging
MWA: migraine with aura
MWoA: migraine without aura
ReHo: regional homogeneity
RFCS: regional functional connectivity strength
ROC: receiver operating characteristic curve
rs-fMRI: resting-state functional magnetic resonance imaging
ViT: Vision Transformer
XAI: explainable artificial intelligence

## Data Availability

The datasets generated or analyzed during this study are not publicly available due to hospital institutional review board regulations but are available from the corresponding author on reasonable request. The implementation details and source code are publicly accessible at our GitHub repository [49].
