# Supplementary material for: Interpretable Artificial Intelligence Analysis of Functional Magnetic Resonance Imaging for Migraine Classification: Quantitative Study
Source: JMIR Med Inform. 2025 Sep 3;13:e72155. doi: 10.2196/72155 (PMC12444220; doi:10.2196/72155)
Supplement: Multimedia Appendix 1 [file medinform_v13i1e72155_app1.doc]

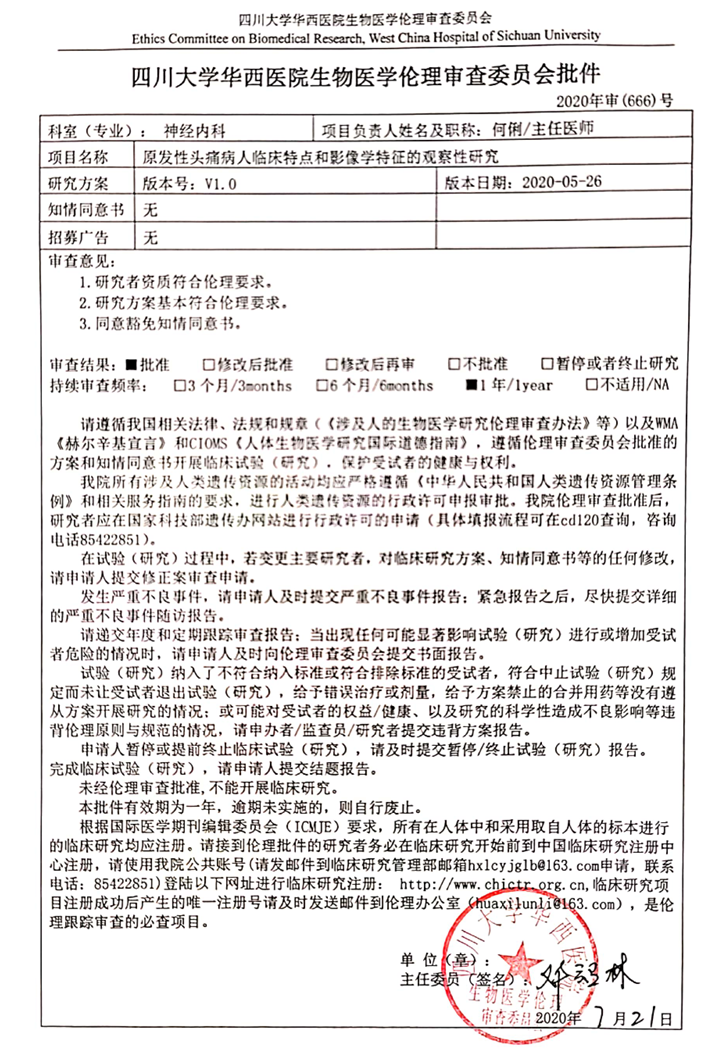


**Fig. S1** The ethical review documentation

**Fig. S2** The results of ViT model

**Table S1** Results of LOOCV validation for the three metrics based on the GoogLeNet model.

| Model | Indicator | Data group | Accuracy | F1-Score |
| --- | --- | --- | --- | --- |
| GoogleNet | ALFF | HC vs. migraine | 85.45% ± 1.21% | 85.33% ± 1.18% |
|  |  | HC vs. MWoA vs. MWA | 84.60% ± 2.95% | 84.42% ± 2.87% |
| GoogleNet | ReHo | HC vs. migraine | 91.30% ± 1.75% | 91.18% ± 1.71% |
|  |  | HC vs. MWoA vs. MWA | 89.95% ± 1.22% | 89.83% ± 1.18% |
| GoogleNet | RFCS | HC vs. migraine | 96.35% ± 1.18% | 96.22% ± 1.20% |
|  |  | HC vs. MWoA vs. MWA | 95.30% ± 2.11% | 95.10% ± 2.09% |
